# Seed Longevity—The Evolution of Knowledge and a Conceptual Framework

**DOI:** 10.3390/plants12030471

**Published:** 2023-01-19

**Authors:** Jayanthi Nadarajan, Christina Walters, Hugh W. Pritchard, Daniel Ballesteros, Louise Colville

**Affiliations:** 1 The New Zealand Institute for Plant and Food Research Limited, Food Industry Science Centre, Palmerston North 4410, New Zealand; 2 USDA—Agricultural Research Service, National Laboratory for Genetic Resources Preservation, Fort Collins, CO 80521, USA; 3 Royal Botanic Gardens, Kew, Wakehurst, Ardingly, Haywards Heath RH17 6TN, UK; 4 Chinese Academy of Sciences, Kunming Institute of Botany, Kunming 650201, China; 5 Faculty of Farmacy, Department of Botany and Geology, University of Valencia, Av. Vicent Estelles s/n, 46100 Valencia, Spain

**Keywords:** seed longevity, cryopreservation, seed ageing, glassy state, cryobiotechnology

## Abstract

The lifespan or longevity of a seed is the time period over which it can remain viable. Seed longevity is a complex trait and varies greatly between species and even seed lots of the same species. Our scientific understanding of seed longevity has advanced from anecdotal ‘Thumb Rules,’ to empirically based models, biophysical explanations for why those models sometimes work or fail, and to the profound realisation that seeds are the model of the underexplored realm of biology when water is so limited that the cytoplasm solidifies. The environmental variables of moisture and temperature are essential factors that define survival or death, as well as the timescale to measure lifespan. There is an increasing understanding of how these factors induce cytoplasmic solidification and affect glassy properties. Cytoplasmic solidification slows down, but does not stop, the chemical reactions involved in ageing. Continued degradation of proteins, lipids and nucleic acids damage cell constituents and reduce the seed’s metabolic capacity, eventually impairing the ability to germinate. This review captures the evolution of knowledge on seed longevity over the past five decades in relation to seed ageing mechanisms, technology development, including tools to predict seed storage behaviour and non-invasive techniques for seed longevity assessment. It is concluded that seed storage biology is a complex science covering seed physiology, biophysics, biochemistry and multi-omic technologies, and simultaneous knowledge advancement in these areas is necessary to improve seed storage efficacy for crops and wild species biodiversity conservation.

## 1. Introduction

The conservation of plant genetic resources is a global necessity, either to implement Green Revolution promises of higher crop yields or to stave off the increasing danger to plant populations brought on by social pressures or the changing climate [1]. Seed storage is the most efficient approach to ensure the availability of plant genetic resources, and the last 50 years have seen the basic question—how long can seeds live?—elevated in importance to meet demands arising in both agriculture and conservation spheres [1]. The reliable prediction of the period for which seeds remain viable (i.e., lifespan or longevity) has been a concern for farmers and seed industries for more than 100 years. In 1908, Ewart created a list of short-, medium- and long-lived seeds stored under ambient environments [2]. Since then, our understanding of factors contributing to seed longevity has increased, and the use of controlled environments to prolong seed viability has led to major advancements in gene banking [1].

Seed moisture content and storage temperature are two critical factors that influence seed lifespan [3,4,5,6,7,8]. Harrington’s ‘Thumb Rules’ estimate that seed life span is halved for each 5 °C increase in seed storage temperature and 1% increase in seed moisture content and that the effects of temperature and moisture are independent and additive. Harrington also cautioned that the first rule does not apply at temperatures below 0 °C or above 50 °C [9]. Modelled additive effects of temperature and moisture are supported by empirical observations conducted at temperatures between 5 °C and 50 °C and relative humidity (RH) between 60% and 90% [10]. It is now understood that as temperature and moisture availability decrease, important physical changes to the seed occur depending on the interacting effects of moisture and temperature [6,11].

The widely used classification of seed storage behaviour (orthodox, recalcitrant and intermediate) was introduced in 1973 (modified in 1991) and provides the basis to understanding why conventional storage conditions are not appropriate for short-lived and desiccation-sensitive seeds [12,13]. Most orthodox seeds are preserved better when they are dried and stored at cold temperatures; based on this knowledge, seed genebank standards were introduced (i.e., storing seeds at −20 ± 4 °C and 15 ± 3% RH, also known as the “conventional method”) [14,15,16].

For many species of concern for conservation, there is no a priori information to predict seed response to storage, and so tools have been developed using environmental cues, seed morphological structure, seed mass, desiccation and temperature sensitivity to predict seed storage behaviour [17,18,19,20]. Recently, the term ‘exceptional species’ was introduced to recognise the need for cryobiotechnology approaches that can be used by genebanks to conserve species suffering from reproductive failure or producing seeds that store poorly using conditions recommended by the FAO [21]. Growing knowledge in the areas of cryobiology and cryobiotechnology provides opportunities to store exceptional species, including highly threatened recalcitrant species [1].

Over the last 50 years, it has been recognised that seed ageing is influenced by the external storage environment (temperature, relative humidity and oxygen) and internal seed features (structure and chemical composition), and such ageing can be detected through physiological, physicochemical, cytological, molecular and genetic changes. Recent advances in understanding seed ageing link vigour decline and viability loss (reviewed in [22]). Oxidative damage caused by reactive oxygen species (ROS) to lipids, proteins and nucleic acids is associated with seed ageing during storage [23,24,25,26,27]. This understanding led to research in developing biochemical markers that could robustly predict viability loss, which can augment traditional germination tests. For small seed lots, non-destructive methods, such as seed volatile quantification and seed thermal fingerprinting, have been proposed for seed viability prediction [28,29,30,31].

This review considers the evolution of knowledge on seed longevity over the past five decades in relation to many facets of the science: the emergence of key research topics in seed physiology, biophysics, biochemistry and multi-omics, greater understanding of the ageing mechanisms, and the development of technologies, including tools to predict seed storage behaviour and non-invasive techniques for seed longevity assessment.

## 2. Seed Storage Classification

Seed storage behaviour is largely dependent on tolerance to desiccation, a trait that is acquired during the latter stages of embryogenesis [32,33,34,35]. Seeds that do not survive desiccation are frequently referred to as ‘recalcitrant’ on account of difficulties in storing them [12]. A larger cross-section of species produces desiccation tolerant seeds, termed ‘orthodox,’ which tend to be more amenable to storage. The orthodox versus recalcitrant delineation provides an immediate perspective on the investment of effort and infrastructure necessary for effective conservation of the two types of seeds in genebanks [1,36,37,38]. In fact, orthodox seeds’ ability to survive storage is the lynchpin in global efforts to conserve plant genetic resources as seeds. The term ‘recalcitrant’ is also used in a horticultural sense to describe seeds that typically exhibit low germination because they have complex requirements for hydration/dehydration and temperature fluctuations before radicles emerge [39,40,41,42,43].

A first assessment of desiccation tolerance usually consists of a measurement of survival (alive or dead) following a single water stress, such as drying to 50% RH [35,44,45,46,47]. Evidence of variation in ‘critical’ water contents or water potentials, which mark lethal drying effects, suggests that desiccation tolerance is actually a continuous phenotype [34,48,49]. Descriptions, such as ‘orthodox with limited desiccation ability’, ‘sub-orthodox’ and ‘minimally recalcitrant’, further suggest overlaps of the two categories [34].

**Figure 1:**
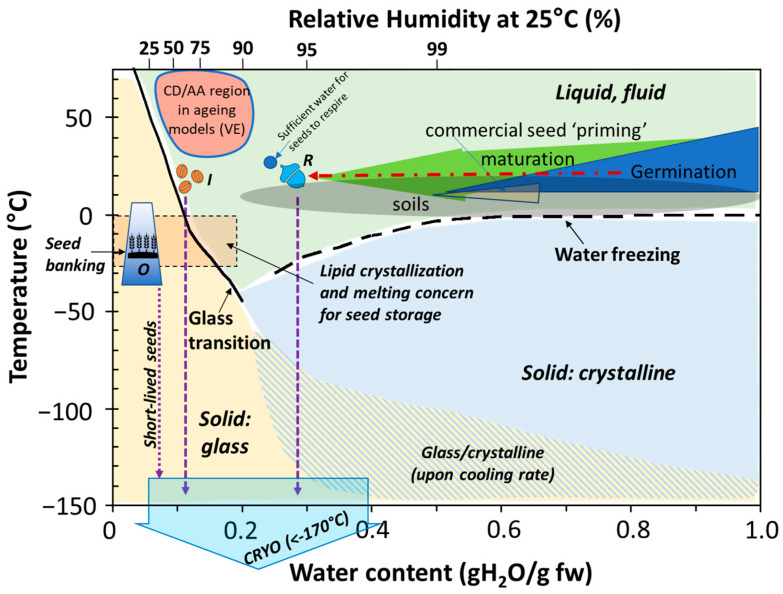
Phase diagram of seed physical states and storage challenges for the different seed storage behaviours. FW: fresh weight; O: orthodox; R: recalcitrant; I: intermediate. CD: control deterioration; AA: accelerated ageing; VE: viability equations; purple arrow: cooling to cryogenic temperatures needed for long-term preservation; red arrow: partial desiccation needed to assure optimal cryopreservation.

**Figure 2:**
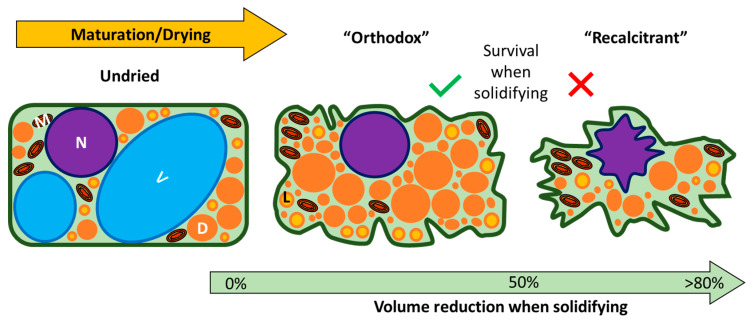
Schematic diagram to illustrate hypothetical differences in cell shrinkage during the drying of orthodox and recalcitrant seed tissues. In orthodox seed cells, dry mass accumulates during maturation and fills the cell volume. Hence, a desiccation force causes only slight cell shrinkage. In contrast, the accumulation of space-filling reserves in recalcitrant seeds is less marked, causing considerable cell shrinkage when a drying force is applied. V, vacuole; N, nucleus; L, lipid body; M, mitochondria; D, other storage reserves, such as plastids filled with starch or protein bodies, or complexes formed by LEA and HSP and raffinose or other oligosaccharides (see, e.g., [22,35]).

The continuous, as well as discrete, aspects of both desiccation tolerance and seed storage categories have launched research relating seed storage behaviour to glass formation. Glass formation, also called vitrification, describes how fluid cytoplasm solidifies when liquid water is removed (Figure 1, temperatures above 0 °C) or when ice formation is prevented (Figure 1, temperatures below −100 °C). Under a high desiccation force (RH < 90%), a dilute mixture, such as hydrated cytoplasm, concentrates and becomes increasingly viscous as the remaining dry components squeeze together. The mixture begins to hold its own shape when dried to RH between 50% and 25% (above 0 °C), resisting further compaction; that is, it solidifies, becoming a glass (yellow shading in Figure 1). Originally, the presence of glasses was hypothesised to confer protection from desiccation, and it was reasoned that glasses form in the cytoplasm of orthodox seeds but not recalcitrant seeds [50,51,52]. Since then, we have learned that the transition from fluid to solid occurs in desiccation-tolerant and sensitive cells alike [53,54]. Rather, it may be the extent of cell shrinkage that occurs before a glass forms that distinguishes recalcitrant and orthodox categories (Figure 2) [22,49]. Cells stocked with dry matter reserves are denser and shrink less, given the same desiccation force, than cells containing a lot of water [47,49,55,56,57] (Figure 2). Literature from the 1970s suggested that cell shrinkage beyond a certain percentage is lethal [58,59]. Once the cytoplasm solidifies, cell shrinkage continues to occur but over an extremely protracted timeframe, something that may be related to the ageing seen in dry orthodox seeds [7,60].

The evolving context of glasses invokes a temporal framework that enhances our understanding of orthodox seed physiology. Orthodox seeds were originally defined in a temporal framework, namely the prolonged shelf life through progressive drying or cooling [5,61]. Harrington’s Thumb Rules [4] and Ellis and Roberts’ Viability Equations (VEs) [8,61] reliably predict shelf-life at moistures and temperatures above the glass transition (Figure 1, dark tan shaded shape between 30% and 90% RH and above 15 °C), but unreliably predict longevity of the glassy cytoplasm [62,63], which forms under the conditions used for genebanking [14]. These empirical models handle moisture and temperature independently; however, we know now that moisture and temperature interact in glasses, as shown in the phase diagram (Figure 1) [64,65]. Differing interpretations of data from empirical models or emerging glassy-state concepts led to a disagreement about the potential benefits versus risks of extreme drying, which are now encapsulated in the literature as ‘ultra-dry technology’ [66]. Importantly, cytoplasmic stability under glassy conditions is likely dependent on cellular composition [22,35,47,49] and is an unexplored frontier for seed storage physiology.

**Figure 3:**
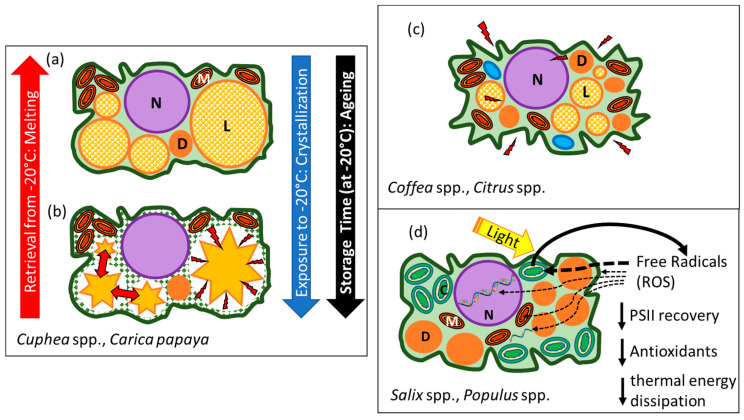
Schematic diagram of the hypothetical dry architecture of ‘intermediate’ seed storage behaviour. (**a**) Cells that survived drying may be damaged in the freezer when (**b**) storage lipids crystallise and leave large gaps in the glassy matrix and increase the potential for oxidative damage or structural collapse. (**c**) Cells that survive sufficient drying to approach cytoplasmic solidification, but die with further drying (represented by some species of coffee or citrus and neem). (**d**) Germplasm presenting increased longevity with decreased storage moisture and temperature, according to models, but having an extremely rapid basal deterioration rate (represented by *Salix* seeds, chlorophyllous fern spores and pollen of many species). Potentially these cells lack protection from oxidative stress, which may be harsher when the light-harvesting photosynthetic apparatus is present. Red arrows and lighting symbols represent areas of physicochemical stress due to drying or lipid crystallisation. C, chloroplasts; N, nucleus; L, lipid body; M, mitochondria; D, other storage reserves such as plastids filled with starch or protein bodies, or complexes formed by LEA and HSP and raffinose or other oligosaccharides (adapted from [22], used with permission from Cambridge Press).

Accepting that both desiccation tolerance and stability of the vitrified cytoplasm are continuous traits, it is not surprising that many seeds do not conform to the simple, dichotomous categories of recalcitrant and orthodox. Shortly after the orthodox–recalcitrant paradigm was accepted, there were reports of exceptions, and a new category, called intermediate, was introduced to accommodate seeds that partially followed empirically derived seed storage models. The first seeds classified as ‘intermediate’ followed model behaviour for moisture, but not for temperature [67,68]. Intermediate seeds stored in the freezer (~ −20 °C) appear to deteriorate faster than those stored in the refrigerator (~5 °C) (e.g., [69]), and this temperature anomaly was linked with the crystallisation of storage lipids (triacylglycerols, TAG) [70] (Figure 3a,b, TAG represented by yellow shapes with a smaller area resulting from crystallisation at subfreezing temperatures). In a material sciences context, dried cells containing TAG can behave like composite materials that become unstable at temperatures at which the constituents have different thermal properties [71] (Figure 3c). The intermediate storage category also includes seeds (e.g., coffee, neem) that survive partial drying—even to the benchmark 50% RH—but then do not survive further drying to conditions allowing cytoplasmic vitrification [67,72] (Figure 3c). Some genebanks also use the intermediate category to group seeds that follow orthodox seed models for moisture and temperature, but viability is not maintained for a long term (decades) under conventional genebank storage conditions [73,74,75,76] (Figure 3d).

The classification of seeds into storage categories was intended to simplify decisions for the efficient conservation of the seeds in genebanks. For example, seeds that exhibit orthodox behaviour can be stored using refrigerators (medium-term) or freezers (long-term) [14] (Figure 1). However, we might wish to cryopreserve orthodox seeds to prolong the shelf life of accessions that are difficult to regenerate (e.g., tree species) or that are relatively short lived [76,77] (Figure 1, purple arrow in “short-lived” seeds). In contrast to the range of options available for orthodox seeds, most seeds exhibiting intermediate or recalcitrant traits require cryogenic storage [36,78,79] (Figure 1, purple arrow for “recalcitrant” and intermediate” seeds). Considerable research activity has focused on categorising species according to the storage physiology of the seed, and this is conveniently summarised in the Seed Information Database [80]. The category of ‘recalcitrant’ is unfortunately named as it conveys the idea that storage is altogether futile, rather than possible if one invests in cryobiological technologies. Terminology that indicates that these seeds are sensitive to desiccation would more likely stimulate the development of cryopreservation methods used for other germplasms, such as shoot tips [79].

The goal of clearly delineating a particular storage behaviour and hence a recommended storage methodology is complicated by observations that (a) populations or collections within a species present varying seed storage behaviours, i.e., seeds of the same species might classify differently depending on growth conditions or genetic lineages [69,70,76]; (b) viability assessments might not accurately reflect potential for full recovery or gradations of damage; and (c) sample sizes required to compare responses to different treatments are prohibitively large compared to seed availability [45,81,82].

A new way to categorise plant species as ‘exceptional’ also conveys the difference in genebanking approaches that use conventional seed storage or cryobiotechnologies [21]. Species producing intermediate or recalcitrant seeds are placed in the exceptional category. In addition, species that exhibit reproductive failure, such as a poor seed set, may be best protected ex situ using clonal propagation and the cryopreservation of vegetative tissues [21].

## 3. Prediction of Seed Storage Behaviour

Henri Poincaré noted that the problem with prediction is that it will only be free from ambiguity as long as the language used is understood [83], and yet the lexicon of seed storage behaviour is incomplete. Moreover, it is important to avoid adopting the simplest theory to enable prediction as it might not adequately explain all the facts under consideration, as J. B. S. Haldane (1927) opined [84].

Following formative work in the 1960s on rice [85], the first comprehensive attempt to formalise the language of seed storage was that of Roberts (1973) [12], who assigned seeds to two main storage classes based on the population response to drying, viz. desiccation tolerant and long-lived (‘orthodox’), desiccation sensitive and short-lived (‘recalcitrant’). Moreover, orthodox seeds benefitted from reducing the moisture content (MC) and temperature in a predictable way, as described through a set of seed viability constants generated under a range of storage conditions [8,80]. The early visualisation of the dependency of dry seed longevity on moisture and temperature was presented as a nomograph, e.g., for onion [86] and barley [87]; that of the latter could predict seed viability after any time in any storage environment within the range −20 to 90 °C and 5–25% MC [87]. This concept was supported by a single viability equation with four constants:

log σ = *K_E_* − *C*_W_log*m* − *C*_H_*t* − *C*_Q_*t*^2^, (1)

such that the distribution of seed death in time (σ) was dependent on the inherent longevity of the species (*K*_E_), the responsivity of longevity to MC (fresh weight basis; *C*_W_) and sensitivity to temperature (*C*_H_*t* and *C*_Q_*t*^2^). One of the main updates to the viability equation was the concept of universality for the temperature constants, *C*_H_ and *C*_Q_ [88]. The mathematical basis for these seed longevity responses, and thus prediction, is that survival curves (viability % plotted against time) are cumulative normal distributions of the negative slope, which become straight lines when viability values are transformed to the probit [89]. Other forms of sigmoidal curve fitting can be used, such as the Avrami equation [62]. The probit transformation enables an estimate of the theoretical initial viability, *K*_i_, based on the back extrapolation of a ‘rapid ageing’ (adverse environment at elevated MC and temperature; dark tan region of Figure 1) survival curve to the intercept at time zero. Accurately determining *K*_i_ may not be possible through a single germination test of 400 seeds; therefore, ‘rapid ageing’ tests are an important component of quality assessment and comparative longevity studies [90,91]. Because *K*_i_ is seed lot-specific and depends on the genotype and pre-storage environment, it also contributes to an understanding of *p*_50_ values, i.e., the time taken for viability to fall to 50%, as under a single set of conditions, a high *K*_i_ seed lot will take much longer to reach *p*_50_ than a low *K*_i_ seed lot. Thus, for mechanistic studies on the genetic control of seed longevity, e.g., homeobox transcription factors and GA in Arabidopsis [92], genome-wide association mapping in barley [93] or rice varieties [94], etc., assessing seed viability after a single ageing time point may not reveal any potential initial differences in *K*_i_. For such studies, it is far better to generate values for *K*_i_, σ and *p*_50_ [91,94]. This is also the case for seed accessions held under long-term storage conditions, but sub-optimal management procedures and insufficient data make reliable estimates of longevity and forecasting regeneration times challenging [95]. The confidence in predicting longevity outcomes can be undermined, particularly when using linear rather than probit models, when there is an uneven density of observations of >85% and <85% viability, in the presence of dormancy, and by a sudden fall in viability or shoulder [27,95]. Such concerns were one of the driving factors to stimulate research on determining RNA degradation [25] or integrity [27] when ageing is asymptomatic to chart and predict the onset of the viability decline, including for dry seeds stored for decades.

Another concern of the viability equation is whether it adequately predicts patterns of viability loss over the longer term (decades or more), when the constants have been generated under environmental conditions that accelerate viability loss (generally months or less), therefore raising the question of whether the seed response under artificial ageing conditions (above Tg, seed dark tan shape in Figure 1) is predictive of the seed response under glassy conditions (below Tg, yellow shading in Figure 1) [96]. Generating the full viability constants consumes much time and seeds, and they are only available for <100 species [80]. Characterising relative seed ageing responses quickly using a single high moisture content at one elevated temperature (artificial ageing [AA]; controlled deterioration [CD]), sometimes combined with elevated oxygen (EPPO), is popular [90,97]. However, assumptions of long lifespans for biodiverse species and the extrapolation from short-term performance to long-term outcomes is not guaranteed [37], as the mechanisms for viability loss in the non-glassy relatively moist state and glassy dry, cooler state will be impacted by the differing structural mechanics and molecular mobility of the system [64]. This may lead to, inter alia, varying patterns of cumulative oxidative stress [98,99]. It is likely that future models of longevity will need to be modified to account for the gaseous environment, e.g., the benefits of anoxia on longevity in the glassy state [97,99].

The low moisture limit of the valid application of the viability equation has long been known; below this limit, increases in longevity are generally not realised [8,96,100]. Similarly, the applicability of the universal temperature constants has limitations below −13 °C, as they could not fully account for the response of *Ulmus carpinifolia* seeds stored at −75 °C, and the quadratic term *C*_Q_*t*^2^ predicts increasingly negative effects of temperature below about −34 °C, for which there is little evidence [8,88]. A logarithmic fit to the effects of temperature on longevity is as good as using the quadratic term [88] and suggests that there is a limiting benefit of extremely low temperature storage but not the damaging one predicted by the quadratic function of the temperature term. Whilst an improvement on −20 °C, longevity at LN temperatures may not be unlimited and is consistent with evidence of a break in Arrhenius behaviour at temperatures well below the glass transition temperature for dry seeds (c. 28 °C) [57,101], perhaps corresponding to the Kauzmann temperature at about −42 °C [101]. It therefore appears appropriate to have alternative prediction models for genebanks that store super dry seeds and to use a viability equation prediction model for commercial seed storage at above 60% RH. In oilseeds, the break in Arrhenius behaviour coincides with a major triacylglycerol phase change between −40 and −7 °C [101] and could explain the problems with ‘intermediate’ seeds, a category of seed storage behaviour identified 30 years ago, e.g., coffee [67]. During rewarming, *Coffea arabica* L. seed lipids start to melt below −20 °C and have a main peak centred at about 5 °C [102]. Seeds of *Cuphea* P.Browne species with similar sensitivity to storage at −18 °C have a main lipid melt temperature ≥ 27 °C; those with melting temperatures < 27 °C are able to tolerate low-temperature exposure [70]. In *Cuphea carthagenensis* (Jacq.) J.F.Macbr. seeds, the rate of deterioration at 5 °C correlates with the rate of TAG crystallisation within the seeds [103], which is also a problem during 12 years of storage at −20 °C for dry spores of the fern *Polystichum aculeatum* (L.) Roth, which have a pre-storage melting peak at around −20 °C [57]. Lipid-related transformations coincide with the poor storage at −18 °C of dry seeds of the orchid *Cattleya aurantiaca* (Bateman ex Lindl.) P.N. Don [synonym of *Guarianthe aurantiaca* (Bateman ex Lindl.) Dressler & W.E. Higgins] [104], and ultra-dry seeds of two species in the Brassicaceae family with poor storage at −5 to −10 °C (i.e., evidence of viability falling within a decade) and a main lipid melt at c. 10 °C [105]. Awareness of the huge variation in seed lipid composition and thermal behaviour, e.g., Brassicaceae [105], led to the suggestion that the optimum sub-zero storage temperature for dry oilseeds could be species-specific [106]. Undoubtedly, the complexity of the sub-zero storage trait for ‘in-between’ species [49] means that future predictive models for seed longevity in the dry state will need to incorporate evidence of cellular volume changes and in oilseeds, account for varying physical states of seed lipids with temperature, including their predilection for crystallisation. This will demand the use of a combination of imaging and biophysical approaches, including thermal fingerprinting.

Predicting seed storage behaviour in ‘orthodox with limited desiccation ability’ (OLDA) [107,108,109], ‘sub-orthodox’ [110], ‘intermediate’ [13,67], ‘Type II’ [106] and ‘in-between’ [49] seeds remains challenging. Nevertheless, numerous means of predicting the binary separation of orthodox vs. recalcitrant seeds have been proposed in the last 30 years (see [111]). Such models are based mainly on trait preferences and frequency estimates for recalcitrant seeds: higher preponderance in the wet tropics and amongst trees and in certain families, larger seed mass, thinner seed coats, less likely to have dormancy, seed/fruit dispersal coinciding (more likely) with peak rainfall, preferring to form seedling banks rather than soil seed banks [112]. The most instructive models for recalcitrant behaviour have relied on ecological correlates, such as: (i) heavier seeded species in Araucariaceae [107]; (ii) multiple criteria keys for certain families (e.g., Meliaceae), using seed weight, moisture content at the time of seed shedding, seed shape and general habitat information [113]; and habitat assessment, with low frequency (≤10%) in the drylands vs c. 50% for tropical moist evergreen forests [18]. An association has also been made between recalcitrance and relatively quick germination [81], facilitated by less resource allocation to protective seed coats (small seed coat ratio). However, tree seed threshold modelling for germination reveals that recalcitrant tree seeds can have longer thermal times than orthodox tree seeds [112]. Nonetheless, the seed coat ratio:seed mass (SCR:SM) probability model for desiccation sensitivity [17] has proven to be valuable in seed storage behaviour studies on species of the wet forests of Australia [114], China [19], Brazil [115] and the Caribbean [116]. The consideration of the taxonomic relationship (congeners, family) and habitat [20], combined with seed mass [117], has also been used to predict the incidence of desiccation sensitivity.

What the predictive models do not yet address is the relative levels of drying sensitivity and variation in hydrated storage times, which differ considerably between recalcitrant seeds of tropical and temperate species. As with ‘intermediate’ seeds and the vast variation in longevity in orthodox seeds, recalcitrant seed responses illustrate that there are multiple syndromes for seed storage behaviour, which means that prediction remains a nascent tool. Future studies should assess the seed storage behaviour of species of key families and from biodiversity hotspots so far under-researched (e.g., MesoAmerica, West Africa, Madagascar, Sundaland, and IndoBurma). In addition, predictive models should also be validated through direct seed biology studies.

## 4. Relationship among Seed Moisture, RH and Temperature

A greater biophysical understanding of the role of water in predicting and extending the shelf life came from integrating concepts developed from food and material sciences [7,50,52,54,64,65]. Early longevity models were based on water content; drying seeds to between 3% (lipid-rich seeds) and 7% (starch-rich seeds) moisture content was the recommended genebank standard in the late 1980s and early 1990s (e.g., [118]). Expressing moisture by the RH used to dry seeds corrected for differences in lipid content and also obviated the need to measure moisture content, which can consume large numbers of seeds [5,11]. Hence revised standards use RH as the preferred metric for moisture status [14]. Many genebanks use a combined term of “equilibrium RH” to emphasise that seeds must dry to a constant mass for the measured RH to be representative of the moisture status.

**Figure 4:**
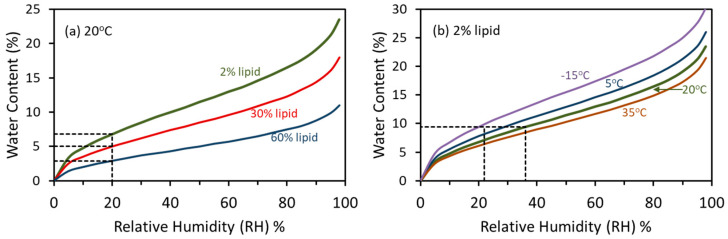
Water sorption isotherms describe the relationship between water content and RH in materials, including seeds. These isotherms were calculated from the Seed Information Database module [80] for seeds at 20 °C and different lipid contents (**a**) and for a seed containing 2% lipid (**b**) at different temperatures. In (**a**), the dashed lines represent water contents associated with 20% RH for seeds containing 2% (e.g., pea), 30% (e.g., lettuce) and 60% (e.g., peanut) lipid: 6.8%, 5.0%, 2.9% water, respectively. In (**b**), dashed lines show the drying conditions needed to meet FAO (2014) standards [14]. That is, storing a seed containing 2% lipid at 20% RH and −15 °C requires adjusting the water content to 9.4% water, which can be done by drying it at 20 °C and 36% RH.

The relationship between water content and RH is described by water adsorption (dry to humid) or desorption (humid to dry; or simply, sorption) isotherms, which appear as reverse-S shaped curves for most orthodox seeds (Figure 4). Typically, water content increases obliquely as RH increases from about 25 to 60%; at lower and higher RH ranges, water content changes abruptly with RH [6,119]. Based on generic isotherms, the RH corresponding to the 3 to 7% water content standard is about 20% at 20 °C, assuming 60 and 2% lipid, respectively ([80]; Figure 4a dashed lines).

As the term ‘isotherm’ implies, temperature affects the amount of water that adsorbs onto molecules, with water content decreasing with increasing temperatures (Figure 4b). The FAO standard for storage at 15–20% RH [14] means that the target water content increases as the storage temperature decreases. For example, 20% RH for a seed containing 2% lipid corresponds to 7.8% or 9.4% water for a seed stored in the refrigerator (5 °C) or freezer (−20 °C), respectively (Figure 4b; [80]).

The 15–20% RH seed storage standard [14] is a conservative bridge between the past and emerging understanding of how water regulates seed lifespans. It stems from the observation of profound changes in the pattern of water regulation of seed shelf life (e.g., the low moisture limit to the VE), which occurs between 20% and 30% RH [5,10,120], as well as problems observed when over-drying and storing seeds below about 10–15% RH [6,11,66]. The recommended storage RH is an improvement of the previous standard, which was based on water content and did not consider composition or temperature interactions. The current standard accepts the idea that the optimum water content for storage increases with a decreasing temperature [6,11,57,120] in a manner similar to the vitrification of cytoplasmic constituents (Figure 1) and further challenges the assumption of the VE that water and temperature have independent effects on longevity.

## 5. Long-Term Conservation and Molecular Motion

How long a seed lot survives with a high level of viability (and vigour) and how long the user needs for it to survive are critical considerations for deciding the storage conditions. Commercial seed lots are expected to survive about 1–5 years (i.e., short term), whilst breeders’ stocks or seeds collected for planned restoration projects are usually expected to survive for 20–25 years (medium term). Preserving genetic resources for agricultural purposes or ex situ conservation requires a longer timeframe again, but exactly how long could depend on numerous factors, such as sample rarity, difficulty of regenerating the sample and the possibility of reintroduction into suitable habitats. Genebanks often target survival durations of about 100 years for these purposes. The immediate question becomes the storage conditions necessary to obtain that long-term conservation goal.

Viability models, described previously, help us to understand the temperature and moisture levels necessary to obtain desired seed longevity for long-term conservation. According to the Seed Information Database SID Seed Viability Constants model, an accession of lettuce (*Lactuca sativa)* seeds that initially germinates at 98–99% will decline to 85% germination after about 69–88 years if stored at −20 °C [80]. This calculation is based on a water content of 5.8%, which is believed to be close to the low moisture limit of reliability for this longevity model at −20 °C. Similar analyses of hundreds of species suggest that the expected life expectancies for high quality accessions (germination > 95%) of most orthodox seeds approach or exceed the 100-year benchmark for long-term storage. Hence, guidelines for the long-term conservation of orthodox seeds recommend storage at –20 ± 4 °C and 15 ± 3% RH [14,15,16]. These conditions are called “conventional” with the use of freezer storage to conserve genetic resources of crops and wild species beginning in the mid-1970s. Early indicators predict that the 100-year goal is both realistic and possible for most species producing orthodox seeds (e.g., [121]); however, there is considerable unexplained variation in longevity within a species, which makes predictions of longevity difficult and necessitates periodic viability testing (international guidelines are every 10 years for seeds expected to be long-lived) [14].

**Figure 5:**
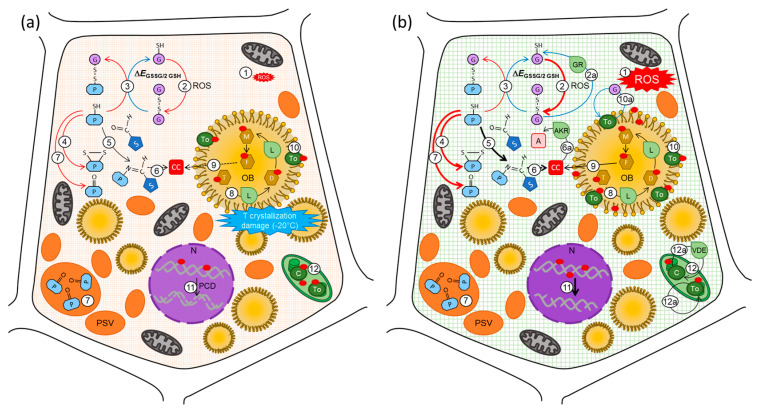
A schematic of hypothesised processes occurring when seeds age under dry (glassy) (**a**) and humid (controlled deterioration (CD)) conditions (**b**) (Figure 1). (**a**): Under low temperature and humidity conditions, the cellular cytoplasm is in a glassy state, which restricts molecular mobility and slows the rate of reactions associated with ageing. The formation of reactive oxygen species (ROS) through auto-oxidative processes occurs at a slow rate. ROS molecules in the atmosphere, by-products of molecular fissures or residuals from energy metabolism when cells were hydrated can diffuse through the glassy matrix depending on the molecular size and pore size (1); ROS accumulation leads to the conversion of glutathione (GSH) to glutathione disulphide (GSSG) and a shift in the glutathione half-cell reduction potential (EGSSG/2GSH) towards more positive values (2). Glutathione can participate in the glutathionylation of Cys residues of proteins (P) (3), which may protect against irreversible oxidation. Intramolecular disulphide bonds are formed (4). The occurrence of Amadori and Maillard reactions between amino acids and sugars (S) has been reported in dry seeds (5) and leads to the formation of a range of reactive and toxic by-products, including carbonyl compounds (CC) (6). Protein carbonylation occurs and storage proteins in protein storage vacuoles (PSV) are particularly susceptible (7). In oil bodies (OBs), higher molecular mobility allows for the lipase (L)-catalysed lipid hydrolysis of triacylglycerols (T), forming diacylglycerols (D), monoacylglycerols (M) and free fatty acids (F) (8). The peroxidation of unsaturated fatty acids of storage and structural lipids leads to the formation of a wide range of radical and reactive by-products, which participate in further reactions, including Maillard reactions (5), and form carbonyl compounds (CC) (9). Tocochromanols (To) scavenge ROS (10) but are not regenerated and thus are gradually depleted, leaving lipids vulnerable to oxidation. At low temperatures, T crystallisation damage may occur (Figure 2b). DNA damage occurs in the nucleus (N), including the internucleosomal cleavage of DNA (laddering) indicative of programmed cell death (PCD) (11). In seeds that retain chlorophyll following maturation, impaired photosystems in the dry state increase oxidation activity (Figure 2d), particularly in the light due to the lack of regeneration of photoprotective compounds, such as carotenoids (C) and tocochromanols (To) (12). (**b**): Under elevated moisture and temperature conditions typically used for CD experiments (e.g., 75% RH and 45 °C), the cytoplasm is in a fluid state, which permits molecular movement and some enzymatic activity. ROS formation occurs at a higher rate than under dry, cold conditions (1). GSH is converted to GSSG (2), but the fluid state of the cytoplasm permits the reduction of GSSG by glutathione reductase (GR) (2a), which limits GSSG accumulation. Cys residues of proteins (P) are glutathionylated (3) and form intramolecular disulphide bonds (4). Further oxidation can lead to the irreversible oxidation of Cys residues. The rate of Amadori and Maillard reactions between amino acids and sugars (S) (5) increases with temperature leading to the formation of reactive carbonyl products (CC) (6). The fluid cytoplasm permits the activity of aldo-keto reductase (AKR) enzymes (6a), which catalyse the detoxification of CC to alcohols (A). Protein carbonylation is increased under CD conditions (7). Lipid hydrolysis (8) and peroxidation (9) lead to damage to storage and membrane lipids. Tocochromanols (To) scavenge ROS (10) and are regenerated by antioxidants (10a), e.g., GSH, so tocochromanol levels are maintained and protect lipids from oxidation. Nucleic acid damage occurs at a higher rate and includes the random fragmentation of DNA in the nucleus (N) (11). Photoprotective and antioxidant capacity is maintained in plastids (12) through the regeneration and synthesis of carotenoids (C), via violaxanthin de-epoxidase (VDE) and tocochomanols (To) (12a). Oxidative reactions are indicated by red arrows and reductive reactions are indicated by blue arrows.

The recommended drying treatment for seed storage [14] is sufficient to change the physical state of the seed cytoplasm from fluid to a non-crystalline (i.e., amorphous) solid also known as ‘glass’ [22] (Figure 1). Molecular mobility and enzymatic activity within solidified cytoplasm are highly limited, and cells become quiescent [22,64,93,99,122] (Figure 5a). Cooling seeds further to seed bank conditions of −20 °C further restricts molecular mobility within the solidified cytoplasm [64]. Currently it is thought that molecular mobility correlates with the rate at which deteriorative reactions occur [57,121,123,124]; this presents an interesting possibility that differences in ageing kinetics among species relate to how molecules move in the glassy structure [57,65].

Life spans of some seeds that tolerate drying and appear to follow the general principles of viability models can be much shorter than the 100-year benchmark [49]. For example, seeds of *Salix* sp. and *Populus* sp. are extremely short-lived, with a greater than 50% loss in viability over four decades reported by conventional seed banks ([76] and references therein). Similarly, seeds of some tree species, such as *Fagus sylvatica* and *Ulmus glabra*, and some species within Poaceae, Apiaceae, Asteraceae and Brassicaceae families have also been reported to show greater than 50% viability loss over four decades [37,77,121]. Cryogenic storage using liquid nitrogen as the cryogen (storage temperature between −170 °C and −196 °C) is recommended [36] and routinely implemented at some seed banks [57,101,125] (Figure 1, purple arrows for “short-lived” seeds). The extreme cold of cryogenic storage is expected to increase longevity by an order of magnitude compared to that with conventional seed storage [88,101]. For example, short-lived seeds of *Salix* sp. and *Populus* sp. retained 80–100% of initial viability when stored in liquid nitrogen for two decades but died within one or two decades when stored conventionally [76]. Similarly, the prediction of average longevity of lettuce seeds was estimated to be nearly 3400 versus 150 years for cryogenic versus conventional storage platforms [101].

There are not many guidelines to assess increased longevity using cryogenic storage compared to conventional storage for orthodox and intermediate seeds. A crude estimate of Q10 ≈ 2.4 (a bit more than doubling the lifespan for a 10 °C decrease in temperature) applies to seeds stored at similar water contents above −20 °C [4,88,101]. However, a much smaller temperature coefficient seems to apply when dried seeds are stored below −20 °C, i.e., under conditions that are far below the point at which the cytoplasm solidifies (Tg) [57,101,123]. With a storage temperature ≤ Tg, long-range motions, such as diffusion, are highly restricted and short-range motions, such as ligand rotations and vibrations, prevail [64,65,123]. Short-range motions are considered important during the ageing of macromolecules [126,127].

Cryogenic temperatures also limit the molecular movement of storage lipids (TAG), which are sequestered as oil droplets in the cytoplasm and form a composite material with the aqueous glassy matrix. TAG tends to crystallise at temperatures below 0 °C, and the extreme cold of liquid nitrogen limits the number of nucleation sites, constraining the crystal morphology [57,102,103,128,129,130,131,132]. Though still conjectural [22], a possible role for TAG crystallisation in oil-rich seeds has been proposed in response to the observed “anomalies” in ageing kinetics at temperatures at which lipid crystallisation is observed [27,68,69,103,104,105]. Seeds containing high proportions of saturated or monounsaturated fatty acids appear more susceptible to temperature anomalies during conventional storage [1,57,70]. Lipid crystallisation has been suggested to be a possible explanatory factor of the “intermediate” storage category (discussed above, Figure 2b). We know that the recrystallisation of lipids into many polymorphic forms can take years, and this may explain the time-dependent expression of temperature anomalies of presumed orthodox seeds during storage under conventional conditions [26].

The long-term conservation of seeds with limited desiccation tolerance (recalcitrant seeds and some intermediate seeds) when compared to that of orthodox seeds requires cryopreservation [36,78,79,102,128,130]. Optimising the water content (between 0.15 and 0.50 g H_2_O g^−1^ dry weight; Figure 1 red arrow) and cooling rate (between 100 °C minute^−1^ and 500 °C second^−1^) is a key strategy to limit the formation of lethal ice crystals [48,102,128,130,133,134,135,136,137] until temperatures allow for cytoplasmic glass transitions ([38,64]; Figure 1, purple arrows for recalcitrant seeds and intermediate seeds).

Optimising the water content and cooling rate may not be sufficient to protect cells during cryo-exposure [134,138,139,140]. Further cryoprotection, using solutions that appear to desiccate the cytoplasm or slow ice formation [141,142,143] and/or solutions that reduce oxidative stress [138,144], appear to increase the survival of non-orthodox seed tissues [134,138]. The vacuum infiltration vitrification method increases the rate of the penetration of cryoprotectants and reduces cytotoxicity [131,145].

Cryobiotechnology is needed to address the existing challenges of conserving genetic resources for the long term. The multifaceted and multidisciplinary approach of cryobiotechnology [146] requires a deep understanding of seed and seed tissue responses to drying and low-temperatures stresses, including why a seed continues to age at liquid nitrogen temperatures, the role of seed storage lipids in ageing and the metabolic recovery responses of embryonic tissues, especially from recalcitrant seeds of a tropical origin [78,79,147].

## 6. Ageing during Storage of Orthodox Seeds

Fifty years ago, it was recognised that seed ageing is influenced by external and internal factors and that could be detected through physiological, cytological and genetic changes. Chromosome damage and gene mutations increase with storage time and are inversely correlated with germination [148,149]. Increases in oxygen tension, temperature and humidity were already known to accelerate ageing [150,151], but it was the gradual accumulation of toxic metabolites that was considered the main cause of ageing [152]. Much of the research on the mechanisms of seed ageing has been conducted on seeds stored at elevated moisture and temperature conditions, known as ‘accelerated ageing’ or ‘controlled deterioration’ [153]. Roberts and Abdalla (1968) [150] showed that storage at 45 °C and a ~18% moisture content resulted in lower chromosome breakage compared to that with storage at a lower temperature or moisture content despite higher viability loss. They concluded that under the most ‘severe’ ageing conditions, other factors were affecting viability, and these factors were insignificant under milder conditions. We now know that these factors reflect the molecular mobility within the cytoplasm, which becomes glassy as the temperature and moisture content decrease, as described previously [64], restricting the movement of molecules and limiting chemical reactions [154]. It is now widely recognised that accelerated ageing and controlled deterioration conditions do not mimic the process of ageing under long term, cold dry storage [37,64,99,155]. Despite this, artificial ageing experiments often provide the only feasible means of studying seed deterioration over a timescale of months, rather than decades, and have contributed greatly to the current state of the knowledge of seed ageing.

Ageing is associated with the accumulation of oxidative damage to lipids, proteins and nucleic acids (Figure 5). Oxidative reactions are promoted by reactive oxygen species (ROS), which originate from the reduction of O_2_, a normal by-product of metabolism or molecular fissures (e.g., Figure 5, reaction 1). ROS levels are controlled by antioxidant enzymes, such as superoxide dismutase, catalase and peroxidases, along with non-enzymatic antioxidants, such as ascorbic acid, glutathione and tocochromanols. Ascorbic acid typically declines during seed maturation, and in the glassy cytoplasm of dry seeds, metabolism and enzymatic activity is restricted, and thus, glutathione, a thiol tripeptide (γ-glutamyl-cysteinyl-glycine), is the main cytosolic redox buffer. During desiccation and storage, glutathione (GSH) is converted to glutathione disulphide (GSSG) (Figure 5, reaction 2). In the dry state, enzymatic regeneration mediated by glutathione reductase (Figure 5b, reaction 2a) is restricted, and thus, glutathione disulphide accumulates. The redox state of glutathione, or specifically the half-cell reduction potential of the glutathione disulphide/glutathione redox couple (*E*_GSSG/2GSH_), is considered a marker of viability [156,157,158,159]. As storage time progresses and seeds age *E*_GSSG/2GSH_ becomes more positive; viability loss is associated with *E*_GSSG/2GSH_ > −160 mV [156]. This threshold appears to be universal across plant, fungal and animal cells [156,160]. More recent comparisons between accelerated ageing, controlled deterioration and ambient and cold storage conditions have shown that *E*_GSSG/2GSH_ shifts to more positive values as seed viability declines, irrespective of the storage conditions [93,98,161] (Figure 5). However, changes in tocochromanol, GSH and GSSG contents follow different patterns under dry and humid conditions (Figure 5b, reactions 10, 10a, 12 and 12a), at a range of temperatures (Figure 5b, reactions 10 and 12), suggesting that the mechanisms of ageing may be different [98,99,161]. At the elevated temperatures and moisture contents (typically 14–18%) used in controlled deterioration experiments, the cytoplasm is in a rubbery or liquid state that increases molecular mobility, permitting enzymatic repair processes and transcription to occur, possibly explaining increases in the tocochromanol content and decreases in the GSSG content during controlled deterioration [99] (Figure 5b, reactions 2a, 10a, 12a). Nevertheless, the oxidative damage sustained during controlled deterioration exceeds the capacity for repair, and thus, viability loss is faster compared to that with ambient or cold storage [98,99,161].

The conversion of thiols to disulphides under more oxidising conditions also affects the thiol groups of cysteine residues in proteins, leading to the formation of intramolecular disulphide bonds, which alter the conformation and function of proteins (Figure 5, reactions 3, 4). Thiol-disulphide conversions are reversible through reduction, catalysed by thioredoxin or glutaredoxin, but the irreversible oxidation of protein thiols to form sulfinic and sulfonic acids can occur under strongly oxidising conditions. The formation of mixed disulphides between cysteine residues of proteins and glutathione, in a process known as *S*-glutathionylation, protects protein thiols from irreversible oxidation and is associated with the maturation drying of orthodox seeds [162].

Protein carbonylation is the irreversible oxidative modification of Lys, Arg, Pro or Thr residues, which flags proteins for proteolysis in hydrated cells. Carbonylation occurs during seed development and germination [163], as well as during seed ageing under controlled deterioration and refrigerated storage conditions [164] (Figure 5, reaction 7). Major targets for carbonylation in Arabidopsis seeds include the 12S cruciferin storage proteins and chaperones. Proteins that are susceptible to oxidation may need protection to ensure their availability during germination [165]. Protein modification can also occur through Maillard reactions between reducing sugars and amino acids under both dry and humid conditions (Figure 5, reaction 5). The relative contributions of lipid peroxidation and sugar hydrolysis to Maillard reactions vary with the seed moisture content and storage temperature [24,154].

Lipid peroxidation during seed ageing has been widely studied, and its association with lost viability is mixed, possibly due to the range of ageing conditions used [23,29,166,167,168,169]. More recent work indicates that lipid oxidation occurs when seeds are stored below 30% RH [30,99,170]. The oxidation and hydrolysis of lipids have been reported in wheat and barley seeds following long-term dry (~6% moisture content) storage at 0 °C and then −18 °C. Non-oxidised storage lipids (TAGs) and structural lipids (phospho- and galactolipids) decrease whilst products of oxidation and hydrolysis, such as mono- and diacylglycerols and fatty acids accumulate as viability declines (Figure 5, reaction 8). The metabolite profiles indicated the involvement of the enzymatic hydrolysis of oxidised lipids [171]. Previously, enzymatic activity has been considered to be minimal in dry seeds due to the low molecular mobility within the glassy cytoplasm. However, Wiebach et al. (2020) [171] suggested that the microenvironment of oil bodies permits the diffusion and catalysis of lipid hydrolysis mediated by lipases.

There is a gradual accumulation of DNA damage, including single- and double-strand breaks, base modification and base loss during seed ageing (Figure 5, reaction 11). The rate of accumulation of damage increases with increasing temperature and moisture, but a base level of damage is apparent in long-term dry seeds. The repair of DNA damage is a critical step during the early stages of imbibition prior to the initiation of cell division [172]. The delayed and decreased uniformity of germination, associated with the loss of vigour in aged seeds, is due to the DNA damage response, which involves a complex signalling network [173]. Irreparable damage leads to the loss of viability through programmed cell death and senescence [26,172] (Figure 5, reaction 11). One of the hallmarks of programmed cell death is DNA laddering, so called because of the regular, nucleosome-sized fragments. DNA laddering occurs in rye seeds with a 10% moisture content, but an increase to 14% moisture content results in random-sized DNA fragments, indicating that the pathways of viability loss are dependent upon the seed moisture content [174]. RNA is more vulnerable to damage than DNA because it is single-stranded. RNA integrity declines with long-term dry storage and damage are detectable before viability is lost. The influence of storage temperature on the kinetics of RNA fragmentation suggests that this reaction is regulated by molecular mobility within a dry seed [27].

There are more than 7.4 million accessions of seed germplasms conserved in gene banks around the world [175]. Ageing with long-term storage leads to the gradual decline of seed vigour and eventual loss of viability, and longevity varies widely among species (reviewed in [37]). Therefore, the periodic monitoring of seed viability of gene bank accessions is required. Germination testing is the standard method for assessing seed viability but is time consuming and destructive. This has led to research into non-destructive and rapid alternatives for monitoring viability [176]. Most non-destructive techniques monitor the early stages of seed imbibition when aged seeds show a delay or impairment in the resumption of metabolism. Microcalorimetry has demonstrated a positive relationship between the rate of metabolic heat production and germination [177] but cannot detect heat flows in the earliest stages of imbibition, and thus, seeds cannot be re-dried and returned to storage. More recently, infrared thermography has shown that differences in the thermal profiles of individual live, aged and dead seeds within the first three hours of water uptake are predictive of germinability [28]. At this early stage of imbibition, it is possible to re-dry the seeds and return them to storage, although subsequent longevity may be affected.

Metabolic activity has also been measured using respirometry, which records oxygen consumption and carbon dioxide production [178]. The Q2 seed analyser enables the automated measurements of oxygen concentrations in sealed vials or wells of a microtiter plate containing individual seeds throughout imbibition. The rate of oxygen consumption has been shown to correlate with germination potential for several species seeds, including tomato, radish and lettuce [179]. However, variation in seed or embryo size, dormancy status, imbibition rate and seed coat permeability affect oxygen consumption. In addition, oxygen consumption within the closed chamber may lead to hypoxic stress and has a negative effect on subsequent germination [180]. Alternative approaches in open systems have used oxygen micro-optodes to measure oxygen influx at the seed surface [181] or flow through respirometry using the infra-red gas analyser of a LI-COR portable photosynthesis system to measure CO_2_ production [178]. However, both are time consuming and lack the convenience of automated, high throughput sampling offered by the Q2 system.

Non-invasive techniques that have been applied to dry seeds include Fourier transform near-infrared (FT-NIR) spectroscopy [182]. This shows high accuracy for sorting individual viable and non-viable seeds based on changes in the seed chemical composition during ageing [183,184]. Whilst FT-NIR spectroscopy collects spectral data from a single point, NIR hyperspectral imaging can provide the spatial resolution of spectral data across a whole sample surface and can scan many seeds together [185].

The analysis of volatile compounds emitted by seeds during storage using gas chromatography-mass spectrometry provides a non-invasive means of probing the reactions occurring during seed storage [30]. Volatile molecules accumulate throughout storage and are detectable prior to the onset of viability loss [170]. Several volatile compounds have been reported to correlate with seed viability across different species and under a range of storage conditions [29,31,186,187,188]; this has raised the possibility of using volatile analysis for non-invasive monitoring of seed viability.

## 7. Conclusions

Over the last 50 years, seed storage science has advanced from anecdotal ‘Thumb Rules’, to empirically-based advances in biochemistry, genomics and biophysics, which have increased the understanding of how seeds can attain cytoplasmic solidification upon drying, how the properties of these intracellular glasses are related to the kinetics of ageing and how seed ageing is characterised by the random and continuous oxidative degradation of proteins, lipids and nucleic acids, which cannot be quenched by the cells’ antioxidant machinery. Seed storage biology is a complex and multidisciplinary science covering seed physiology, biophysics, biochemistry and multi-omic technologies (genomics, transcriptomics, proteomics, metabolomics, ionomics and phenomics). Knowledge advancement and technology development in these fields have helped us to better understand and predict seed storage behaviour and to optimise the storage environment that prolongs viability during storage, supporting agriculture, ex situ conservation and the sustainable use of seeds.

## Data Availability

Not applicable.
